# Anti-angiogenic therapies in cancer: from endogenous inhibitors to bispecific VEGF x PD-(L)1 antibodies

**DOI:** 10.3389/fimmu.2026.1736806

**Published:** 2026-01-23

**Authors:** Luis Álvarez-Vallina, Laura Sanz

**Affiliations:** 1 CNIO-HMRIB Cancer Immunotherapy Clinical Research Unit, Spanish National Cancer Research Centre (CNIO), Madrid-Hospital del Mar Research Institute Barcelona (HMRIB), Madrid/ Barcelona, Spain; 2 Banc de Sang i Teixits, Barcelona, Spain; 3 Molecular Immunology Unit, Biomedical Research Institute Hospital Universitario Puerta de Hierro-Segovia de Arana Majadahonda (IDIPHISA), Madrid, Spain

**Keywords:** angiogenesis inhibition, antibody engineering, bispecific antibodies, cancer immunotherapy, monoclonal antibodies

## Abstract

Based on the hypothesis that neovascularization was required for tumor growth, the search for angiogenesis inhibitors attracted considerable attention, leading to the development of the monoclonal antibody bevacizumab against vascular endothelial growth factor (VEGF) which is currently standard treatment in several types of cancer. However, and despite encouraging preclinical data, clinical trials frequently failed to translate into benefits for patients due to limited efficacy, resistance and toxicity. Resistance mechanisms include triggering of alternative proangiogenic pathways, non-angiogenic vascularization, and the unforeseen heterogeneity of tumor endothelial cells. Early efforts to disrupt key interactions between extracellular matrix and endothelial cells via integrin or metalloproteinase inhibitors also had modest clinical outcomes, mainly due to poor selectivity and compensatory mechanisms. Similarly, the promise of endogenous angiogenesis inhibitors like endostatin and angiostatin did not led to durable clinical responses. Recent studies have shown that VEGF contributes to immune suppression, and therefore anti-VEGF therapy can not only normalize vasculature, improving immune infiltration, but also help to reshape tumor microenvironment. This has led to successful combinations of antiangiogenic agents and immune checkpoint inhibitors, now approved in several indications, including renal cell and hepatocellular carcinomas. Based on these results, bispecific antibodies targeting simultaneously VEGF and PD-(L)1 are emerging as promising therapeutic agents, with several worldwide phase 3 trials ongoing. Globally, around twenty bispecifics and trispecifics are in clinical development. In this review, we recapitulate previous successes and failures of anti-angiogenic strategies, and explore the potential of VEGF x PD-(L)1 antibodies as a new paradigm in cancer treatment.

## Introduction

In 1971 Judah Folkman published a seminal paper proposing that tumors could starve without adequate blood supply; therefore, inhibition of tumor neoangiogenesis could be a promising therapeutic strategy (1). Despite initial skepticism, this concept gained recognition over time and was eventually declared a hallmark of cancer (2). In 2004 the first anti-angiogenic therapy, the anti-vascular endothelial growth factor (VEGF) monoclonal antibody (mAb) bevacizumab, was approved by the U.S. Food and Drug Administration (FDA). At that time, angiogenesis inhibition was actively pursued by many research groups, including ours. Early approaches included blocking pro-angiogenic growth factors, upregulation of endogenous inhibitors or disruption of interactions between tumor endothelial cells (TEC) and their supportive extracellular matrix (ECM) by targeting the integrins implicated in such interactions, proteases responsible for ECM remodeling or specific ECM components. Despite encouraging results in preclinical models, many candidates failed in clinical trials, and even approved therapies fell short to expectations due to limited efficacy and the emergence of drug resistance.

Here, we provide an overview of the development of anti-angiogenic strategies and the challenges they faced to become effective cancer treatments. We also discuss new avenues for the development of therapies with improved efficacy, mainly combinations with immune checkpoint inhibitors (ICI) such as mAb against programmed cell death protein 1 (PD-1) or PD-1 ligand, or more recently, development of anti-VEGF x anti-PD-(L)1 bispecific antibodies. Anti-angiogenic agents aimed to the treatment of ophthalmic conditions are not addressed here; for an in depth account of the angiogenic process role in cancer and other diseases we recommend excellent reviews recently published (3, 4).

## Inhibition of angiogenic growth factors: success and limitations

**Figure 1:**
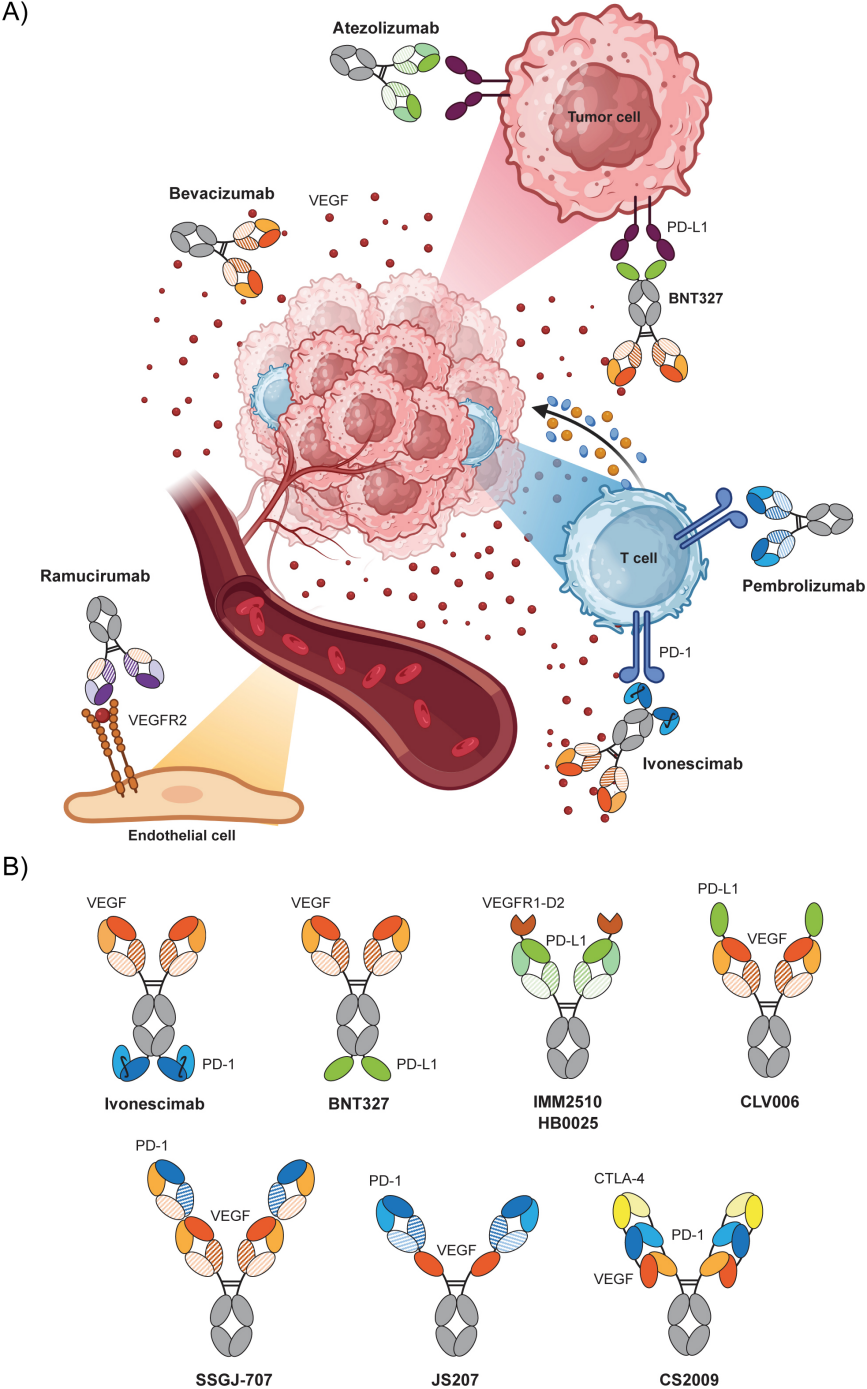
**(A)** Comparative mechanism of action of monospecific vs bispecific antibodies against VEGF/VEGFR and PD1/PDL-1 axes and their targets. **(B)** Schematic representation of some of the multispecific antibodies VEGF x PD-(L)1 currently in clinical trials. Anti-VEGF binding domains are depicted in orange, anti PD-1 in blue and anti-PD-L1 in green. Bi-or trispecific molecules are obtained by adding extra antibody fragments (in Fab, scFv or VHH formats) or a VEGFR extracellular domain.

The human VEGF family is comprised of five related glycoproteins: VEGF-A, VEGF-B, VEGF-C, VEGF-D and placental growth factor (PIGF), which interact with three receptor tyrosine kinases (TK): VEGF receptor (VEGFR)-1, VEGFR-2 and VEGFR-3. Among them, the VEGF-A/VEGFR-2 axis plays a central role in both physiological and pathological angiogenesis, promoting proliferation, migration and differentiation of ECs (5). Indeed, most angiogenesis inhibitors currently approved in cancer are either mAb against VEGF-A or VEGFR-2 (**Figure 1A**), or small-molecule VEGFR tyrosine kinase inhibitors (TKI) (6).

The anti-VEGF mAb bevacizumab is used in the therapeutic management of metastatic colorectal cancer (CRC), combined with chemotherapy in either first-line or second-line treatment (7), as well as in a number of other solid tumors. In 2014, ten years after the approval of bevacizumab, the anti-VEGFR-2 mAb ramucirumab hit the market for the treatment of gastric cancer (8). Intriguingly, and despite the exponential increase in antibody-based therapies for cancer (9), this was the last monospecific antiangiogenic antibody to gain regulatory approval for over a decade. In July 2025, the China National Medical Products Administration (NMPA) cleared the anti-VEGF mAb suvemcitug, with superior activity compared to bevacizumab (10), for the treatment of platinum-resistant ovarian cancer (11).

On the other hand, at least ten small-molecule VEGFR TKI including sunitinib (the first approved), pazopanib and axitinib are widely used in the treatment of different tumors (6). However, their clinical use is frequently associated with cardiovascular adverse events due to poor selectivity, since they inhibit a broad spectrum of tyrosine kinases beyond VEGFR (3). In November 2023 the FDA approved fruquintinib for the treatment of mCRC, a VEGFR-1,-2,-3 inhibitor which belongs to a new class of TKI with higher selectivity and decreased risk of off-target toxicity (12).

Regardless of their mechanism of action, therapeutic resistance to VEGF/VEGFR inhibitors appears in most types of cancer. This was initially unexpected, given the genetic stability of EC compared to malignant cells, which was supposed to prevent the development of resistance. Different mechanisms underlie this phenomenon, including upregulation of alternative proangiogenic factors beyond the VEGF axis, such as PlGF and fibroblast growth factor. In addition, tumors can rely on non-angiogenic mechanisms to secure blood supply. Alternative modes of vascularization not susceptible to VEGF/VEGFR inhibitors include vasculogenic mimicry, whereby tumor cells acquire endothelial cell (EC)-like functions (13, 14) and vascular co-option, wherein tumor cells migrate along preexisting blood vessels (15). Finally, a factor not fully appreciated in early studies is the own heterogeneity of TEC (16). In 2000, St Croix et al. published early bulk RNA-sequencing (RNA-seq) data of EC from normal and malignant colorectal tissues, revealing a distinct transcriptome signature in TEC (17). However, it would take another two decades before single-cell RNA-seq (scRNA-seq) studies uncovered the true extent of EC heterogeneity across healthy tissues and tumors, including differential sensibility to VEGF blockade. A seminal scRNA-seq study in lung cancer by Goveia et al. showed unexpectedly that less than 10% of TECs had angiogenic signatures, and were therefore susceptible to classic anti-angiogenic therapies (18). These low numbers of targetable cells could explain, at least in part, resistance to anti-VEGF therapies. Subsequent scRNA-seq studies in breast cancer have further characterized TEC subpopulations (19). Interestingly, scRNA-seq has revealed a largely similar transcriptome shared by co-opted, quiescent tumor EC and pericytes and their normal counterparts, underscoring that co-opted vascular cells differ from the angiogenic ones (20).

## Blocking interactions with the extracellular matrix

The ECM plays an essential role not only in tumor angiogenesis (21) but also in the tumor biology itself, modulating neovascularization as well as cancer cell intravasation and extravasation (22) and even immune evasion (23). Unfortunately, therapeutic strategies targeting regulators of cell-ECM interactions, including integrins and matrix metalloproteinases, have thus far failed to achieve clinical success in oncology.

Integrins expressed by TECs have been extensively investigated, being αvβ3 (upregulated in angiogenic ECs) probably the most studied. Indeed, inhibitors of αvβ3 demonstrated encouraging therapeutic effects in preclinical models. However, anti-αvβ3 blocking antibodies (such as vitaxin/MEDI-522) tested in patients with solid tumors failed to demonstrate clinical benefit, and other pan-αv antibodies, such as intetumumab/CNTO 95, followed the same path (24). Selective targeting of integrins to halt tumor angiogenesis remains challenging, probably due to their overlapping expression pattern and subsequent compensation mechanisms (25).

Integrin αvβ3 interacts with the arginine–glycine–aspartate (RGD) sequence present in many ECM proteins, therefore RGD-based antagonists were initially expected to exert effects similar to those of anti-integrin mAbs. Cilengitide, a cyclic RGD peptide which also inhibits αvβ5 and α5β1, failed to improve OS in Phase 3 trials (26). Paradoxically, RGD-mimetics could act as agonists at low concentrations and enhance angiogenesis (27), explaining at least in part the clinical failure of cilengitide. More recently, combinations of low-dose cilengitide with the chemotherapeutic agent doxorubicin have been used *in vivo* with improved drug delivery (28).

The interest of integrin-modulating therapies in cancer has declined, but there is still a niche for them in vascular, inflammatory and fibrotic diseases, with some agents already in the market taking advantage of the lessons from previous oncology-focused clinical trials (29).

## Direct targeting of ECM components

Other strategies have focused on finding potential markers for the targeted delivery of therapeutic agents to angiogenic vasculature. Probably the best known of such markers is an alternatively spliced segment of fibronectin, the ED-B domain, which is restricted to sprouting blood vessels. The single-chain variable fragment (scFv) L19, which binds ED-B with high affinity, has shown neovasculature targeting *in vivo* (30) and antibody–cytokine fusion proteins have been developed for cancer therapy. Notably, combination of the immunocytokines L19-IL2 and L19-TNF in patients with locally advanced melanoma has recently demonstrated clinical benefit in a Phase III study (31).

However, the use of the ECM itself as a therapeutic target has remained largely unexplored. We focused on laminin, a major basement membrane component with a well-known role in angiogenesis, and selected several laminin-specific antibody fragments (32). Among them, the scFv L36 was functionally active, inhibiting angiogenesis and tumor growth *in vivo*. Based on the identification of laminin binding sites required for EC migration and vessel maturation, we proposed that disruption of this interaction was responsible for the therapeutic effect of L36 (33). However, clinical development was not pursued further due to unfavorable pharmacokinetics resulting from scFv small size and short serum half-life.

## Matrix metalloproteinase inhibitors

Matrix metalloproteinases (MMP) constitute an extense family of more than 20 endopeptidases that control ECM remodeling and regulate multiple steps of the angiogenic process (34). Among them, MMP-2 and MMP-9 have been specifically involved in the onset of tumor angiogenesis (the “angiogenic switch”) (35, 36). Despite promising preclinical data, all Phase 3 trials with MMP inhibitors failed in reducing tumor burden or improving OS (37). In addition, broad spectrum inhibitors were often associated with significant toxicity due to poor selectivity. In fact, some MMP are essential for tumor progression, but others play host-protective functions. The development of specific MMP inhibitors has been hampered by the high homology between MMP and the difficulty in identifying specific substrates (38). Subsequently, focus shifted toward the development of targeted inhibitors, such as the anti-MMP-9 andecaliximab, which showed clinical activity without toxicity in Phase 1 trials but failed to provide efficacy in a Phase 2 trial in gastric adenocarcinoma (39). More recently, next-generation MMP inhibitors with improved toxicity profiles have emerged as promising drug candidates for the treatment of different conditions, but the fact is that no one has been marketed for cancer after decades of research (40).

## Endogenous inhibitors of angiogenesis

In the 90s and early 2000s, the concept that the angiogenic switch in tumors was due to an imbalance between pro- and anti-angiogenic factors led to the characterization of more than 20 different endogenous inhibitors of angiogenesis, many of them small fragments of larger proteins (41). Such inhibitors would restore the balance with a low toxicity profile, since they were naturally produced in both physiological and pathological conditions. Among the most studied were angiostatin, a fragment of the serum protein plasminogen (42), and endostatin, derived from the C-terminal NC1 domain of collagen XVIII (43). Both were reported to suppress the growth of different tumors in mice, with cycled treatment of tumor-bearing mice with endostatin leading to durable responses and, in some cases, complete tumor growth arrest. Unfortunately, endostatin ended up as the most notorious example of unfulfilled promise in the field of anti-angiogenic therapies, and concerns were raised about reproducibility of preclinical results (44). In parallel, Phase I studies of human endostatin in patients with advanced solid tumors showed limited antitumor activity (45) or directly absence of clinical responses (46). In a second life for endostatin, an independently developed variant with nine extra amino acids and enhanced stability was approved by NMPA in 2005 for the treatment of non-small cell lung cancer (NSCLC), and several clinical trials are ongoing (47). Nevertheless, the potential molecular mechanisms mediated by endostatin are still not clear, and issues regarding poor pharmacokinetics and problems in the production of the active protein need to be addressed (48).

Amid the enthusiasm surrounding endostatin, we engineered a fusion protein consisting of the full length NC1 domain of collagen XVIII and the previously reported anti-angiogenic antibody fragment L36, with only a modest increase in its therapeutic effect attributable to endostatin (49). Unexpectedly, the trimerization domain located at the N-terminus of the NC1 domain proved to be a valuable structural element for generating multivalent, mono- or bi-specific antibodies, which were subsequently termed “trimerbodies” (50, 51). Notably, a trimeric bispecific 4-1BB agonist antibody targeting EGFR induced robust antitumor immunity without systemic toxicity in preclinical models (52), highlighting the platform’s translational potential, still in debt with endostatin.

## The next step: combination with immune checkpoint inhibitors

Different studies have identified novel properties of TEC as modulators of the immune response and demonstrated its contribution to the immunosuppressive TME (19, 53). Indeed, “anergic” TEC can impair T-cell tumor infiltration via the downregulation of adhesion molecules such as vascular cell adhesion molecule-1 (VCAM-1) and intercellular cell adhesion molecule-1 (ICAM-1) (54, 55). Importantly, the anti-PD-L1 mAb atezolizumab in combination with bevacizumab enhances antigen-specific T-cell migration in metastatic renal cell carcinoma (56). Classic studies of resistance to immunotherapy have focused on antigen presentation and interferon signaling pathways as critical mediators, but the list keeps growing. For example, increased VEGF expression can inhibit dendritic cell maturation and promote the expansion of immunosuppressive cell subsets within the TME (57). In this line, a recent study has revealed that targeting VEGF along with PD-L1 and CTLA-4 blockade in a murine model of cholangiocarcinoma promotes rewiring of T regulatory cells, with a crucial role in the immunosuppressive TME, toward an anti-tumoral T helper-1 cell-like “fragile” state (58).

Another factor contributing to ICI resistance is hypoxia, a condition frequently found in the TME due to the insufficient oxygen supplied by abnormal blood vessels to rapidly growing tumors. Hypoxic conditions can lead to metabolic reprogramming of tumor cells, inhibition of apoptosis and increased invasiveness; on the other hand, to the expression of factors like VEGF and TGF-β which inhibit the cytotoxicity, proliferation and infiltration of T cells (59). Indeed, hypoxia was identified as a key feature in a lung cancer mouse model of acquired resistance to ICI, with T cells excluded from hypoxic tumor regions, and a hypoxia signature generated from scRNA-seq data was associated with decreased progression-free survival (PFS) in a cohort of NSCLC patients treated with anti-PD-1/PD-L1 mAb (60). Moreover, targeting hypoxic regions with a hypoxia-activated cytotoxic agent delayed the onset of resistance to ICIs in this murine model.

Based on these considerations, there is a strong rationale for the use of antiangiogenic drugs in combination with ICI (**Figure 1A**). Obviously, the clinical benefits will depend on the net balance between the two potential outcomes of vascular remodeling by VEGF blockade (61). On one hand, tumor vessel pruning can exacerbate hypoxia and foster the hostile TME; conversely, vascular normalization can increase lymphocyte infiltration and activation, and decrease the number and function of inhibitory immune cells. If these opposing effects do not temporally overlap, optimizing treatment sequencing will be crucial for the success of combination regimens. Mathematical models suggest that concurrent administration of anti-VEGF and anti-PD-(L)1 antibodies would be optimal if the first induces vessel normalization and increases vessel perfusion in tumors, whereas a sequential regimen would be preferable if VEGF inhibition reduces tumor vessel perfusion (62). Indeed, short-course anti-VEGF treatment, unlike chronic exposure, has been shown to transiently normalize aberrant vasculature, potentially improving ICI biodistribution within poorly perfused tumors (63). Whether these combined therapies would act through additive or synergistic mechanisms remains an open question.

In this context, several phase III trials have demonstrated the clinical benefit of combined treatments, leading to at least seven regulatory approvals to date (13). For example, combinations of the TKI axitinib with either pembrolizumab (anti-PD-1) or avelumab (anti-PD-L1) have significantly improved OR rates by up to 55% and are now standard first-line treatments for advanced renal cell carcinoma. Similarly, the combination of atezolizumab plus bevacizumab has become the new benchmark for first-line therapy in advanced hepatocellular carcinoma (64). In 2021, the FDA approved atezolizumab plus bevacizumab and chemotherapy in first-line metastatic NSCLC, with or without EGFR genomic alterations, based on the results of the IMpower150 study (NCT02366143) (65). However, findings from IMpower151 (NCT04194203) trial, conducted in China to address regional differences, are inconsistent with the significant PFS and OS improvements observed in IMpower150, and reasons for these geographical differences remain to be analyzed in depth (66). Similarly, some VEGFR TKI plus ICI combinations have not demonstrated a clear advantage over docetaxel in patients with advanced NSCLC who had progressed on both chemotherapy and an ICI (67).

## A change of paradigm: anti-VEGF x anti-PD-(L)1 bispecific antibodies

The clinical success of anti-VEGF and ICI combinations provides a strong rationale for the development of bispecific (bsAb) and even trispecific antibodies targeting simultaneously VEGF and PD-(L)1 (**Figure 1A**). Intriguingly, all of bi- and trispecifics depicted in **Figure 1B** are symmetric and tetravalent (bispecifics) or hexavalent (trispecifics), fragment-based antibodies or fusion proteins. In contrast with most bispecifics in the market, such as T cell engagers, which tend to be asymmetric, bivalent IgG-like antibodies which require heavy chain heterodimerization technologies for proper assembly. On one hand, probably this shift aims to simplify the design and the production process. On the other, increased valency of these molecules implies a gain in avidity, and therefore in functional affinity with respect to monovalent binders. How the subtle differences among the formats represented here will influence their therapeutic potential is difficult to foresee.

Currently, approximately 20 such antibodies are under clinical evaluation, mostly in early-phase trials (**Table 1**, **Figure 1B**). This dual targeting approach may be superior to the individual administration of two individual monospecific mAb, while reducing cost and formulation complexity. The most advanced in clinical development is ivonescimab (AK112/SMT112), a tetravalent bispecific construct consisting of an anti-VEGF IgG with two anti-PD-1 scFv appended to the C-terminal ends of heavy chains, which has demonstrated cooperative binding to each target and enhanced blockade of both signaling pathways (68, 69). Of note, ivonescimab outperformed the anti-PD-1 pembrolizumab as first-line monotherapy in a head-to-head clinical trial HARMONI-2 of NSCLC (NCT05499390), nearly doubling PFS (median of 11,1 months compared to 5,8 months with pembrolizumab) (70), although confirmatory studies are needed to establish OS benefit (71, 72). In May 2024, ivonescimab in combination with chemotherapy received its first approval in China for the treatment of patients with EGFR-mutated advanced non-squamous NSCLC who have progressed after TKI therapy (73). The approval was based on results from the randomized, double-blinded Phase 3 HARMONi-A study (NCT05184712), which demonstrated a significantly improved PFS (74) and recently reached the OS clinical endpoint. In Apr 2025, NMPA approved ivonescimab for the first-line treatment of PD-L1+ advanced NSCLC without EGFR or ALK mutations, and three months later as a first-line treatment for squamous NSCLC. The first global Phase 3 trial to evaluate ivonescimab plus chemotherapy (HARMONi) (NCT06396065) in a post-EGFR TKI NSCLC setting showed an increase in PFS consistent with the HARMONi-A study and a positive trend in OS. Another two global Phase 3 studies are ongoing for the first-line treatment of NSCLC patients comparing ivonescimab versus pembrolizumab, HARMONi-3 (NCT05899608) and HARMONi-7 (NCT06767514), the latter in patients with PD-L1 expression ≥50%.

**Table 1:** VEGF x PD-(L)1 multispecific antibodies in clinical trials (clinicaltrials.gov; last accessed on Dec 27, 2025).

Drug name	Specificity	Clinical trial	Phase	Indication	Status	Last update	Sponsor	Partner	Ref
Ivonescimab (AK112/SMT112)	VEGFxPD-1	NCT05184712 NCT05499390 NCT06396065 NCT06767514 NCT05899608 NCT07228832	3 3 3 3 3 3	NSCLC NSCLC NSCLC NSCLC NSCLC CRC	Active, not recruiting Active, not recruiting Active, not recruiting Recruiting Recruiting Recruiting	28/06/2024 05/03/2025 08/10/2024 25/11/2025 26/11/2025 14/11/2025	Akeso Akeso Summit Summit Summit Summit	Licensed to Summit (2022)	(68–74)
PF-08634404/ SSGJ-707	VEGFxPD-1	NCT06980272 NCT07222800 NCT07222566 NCT07226999	3 3 3 2/3	NSCLC CRC NSCLC SCLC	Recruiting Recruiting Recruiting Not yet recruiting	24/06/2025 30/10/2025 30/10/2025 12/11/2025	3SBio Pfizer Pfizer Pfizer	Licensed to Pfizer (2025)	(75, 76)
JS207	VEGFxPD-1	NCT06969027 NCT06954467	2 2	NSCLC HCC	Recruiting Recruiting	04/07/2025 02/07/2025	Junshi Bioscience		(77)
MK-2010/LM-299	VEGFxPD-1	NCT06650566	1/2	Solid tumors	Recruiting	02/05/2025	LaNova	Licensed to Merck (2024)	
MHB039A	VEGFxPD-1	NCT06345482	1/2	Solid tumors	Active, not recruiting	20/11/2025	Minghui Pharma		
SCTB14	VEGFxPD-1	NCT06304818	1/2	Solid tumors	Not yet recruiting	15/03/2024	Sinocelltech		
AI-081	VEGFxPD-1	NCT06635785	1/2	Solid tumors	Recruiting	10/11/2025	OncoC4		
RC148	VEGFxPD-1	NCT06883630	1	NSCLC	Recruiting	15/07/2025	RemeGen		
CS2009	VEGFxPD1xCTLA-4	NCT06741644	1	Solid tumors	Recruiting	16/09/2025	CStone Pharma		(86)
HC010	VEGFxPD-1xCTLA-4	NCT06307925	1	Solid tumors	Recruiting	13/05/2025	HC Biopharma		(87)
GB268	VEGFxPD-1xCTLA-4	NCT06934616	1	Solid tumors	Not yet recruiting	30/04/2025	Genor Biopharma		(88)
BNT327/PM8002/BMS986545 (pumitamig)	VEGFxPD-L1	NCT06712355 NCT06712316 NCT06419621 NCT06616532 NCT07221357	3 2/3 3 3 2/3	SCLC NSCLC TNBC SCLC CRC	Recruiting Recruiting Recruiting Recruiting Recruiting	26/11/2025 03/10/2025 12/03/2025 19/12/2024 18/12/2025	BioNTech BioNTech Biotheus Biotheus BMS	Biotheus acquired by BioNTech (2024). Deal to co-develop BNT327 with BMS (2025)	(79, 80)
B1962	VEGFxPD-L1	NCT06838546	2	CRC	Not yet recruiting	20/02/2025	Tasly Biopharma		
IMM2510 (palverafusp alpha)	VEGFxPD-L1	NCT06746870 NCT07170787	2 1/2	NSCLC TNBC Solid tumors	Not yet recruiting Not yet recruiting	24/12/2024 12/09/2025	ImmuneOnco Biopharm	Licensed to Instil Bio (2024)	(81, 82)
HB0025 (sotiburafusp alfa)	VEGFxPD-L1	NCT06758557	1	NSCLC, endometrial carcinoma	Recruiting	23/01/2025	Huabo Biopharm		(83, 84)
CVL006	VEGFxPD-L1	NCT06621615 NCT07157956	1 1/2	Solid tumors Solid tumors	Not yet recruiting Not yet recruiting	01/10/2024 05/09/2025	Convalife Pharma		
AP505	VEGFxPD-L1	NCT06723964	1	Solid tumors	Active, not recruiting	09/12/2024	AP Biosciences		
DR30206	VEGFxPDL1xTGF-β	NCT06132828	1	Solid tumors	Recruiting	03/07/2024	Zhejiang Doer		(89)

CRC, colorectal cancer; HCC, hepatocellular carcinoma; NSCLC, non-small cell lung cancer; SCLC, small cell lung cancer; TNBC, triple-negative breast cancer.

Other VEGFA x PD-1 bsAb with a variety of formats (**Figure 1B**) are in Phase 2/3 of clinical development. SSGJ-707/PF-08634404 is another tetravalent antibody based on the CLF^2^ (common light chain linear-Fabs-IgG) platform (75), which has demonstrated a 10-fold increase in PD-1 binding affinity compared to ivonescimab in the presence of VEGF (76). A large Phase 3 clinical trial of PF-08634404 versus pembrolizumab as first-line treatment for PD-L1+ advanced NSCLC (nonsquamous and squamous) has just been launched (NCT06980272). Another phase 3 study will test PF-08634404 in CRC (NCT07222800), and several other clinical trials are ongoing (**Table 1**).

LM-299 comprises an anti-VEGF antibody linked to two C-terminal single domain anti-PD-1 antibodies; a Phase 1/2 clinical trial is also enrolling patients with advanced solid tumors (NCT06650566). JS207 consists of an anti−PD−1 mAb with an anti−VEGFA VHH embedded between the hinge and Fc regions of both heavy chains (77). At least three Phase 2 clinical trials with JS207 are recruiting NSCLC (NCT06022250), CRC (NCT06885385) and HCC (NCT06954467) patients.

The use of an anti-PD-L1 antibody fragment instead of an anti-PD-1 binder might restrict VEGF neutralization to the PD-L1-overexpressing TME, increasing local concentration of the bsAb in the tumor tissue over healthy tissue and limiting systemic exposure (78). This strategy is exploited by BNT327/BMS986545/pumitamig, a VEGFA x PD-L1 bsAb with a format similar to that of LM-299 (an anti-VEGF mAb with two appended VHH) (**Figure 1B**). There are two ongoing global Phase 3 and Phase 2/3 clinical trials: Rosetta-Lung-01 *(*NCT06712355) in extensive-stage SCLC (ES-SCLC) (79) and Rosetta-Lung-02 (NCT06712316) in NSCLC (80), along with another two China-only Phase 3 trials in TNBC (NCT06419621) and SCLC patients (NCT06616532). Rosetta-Lung-02 was the second global phase 3 trial launched to compared a VEGF x PD-(L)1 bispecific with pembrolizumab in first-line NSCLC, after HARMONi-3 and before the PF-08634404 study. Similarly, these three bispecifics (ivonescimab, pumitamig and PF-08634404) have just initiated phase 3 clinical trials in CRC testing a chemotherapy combination against bevacizumab plus chemotherapy (**Table 1**).

A different approach is shared by two fusion proteins, IMM2510/AXN-2510 (81, 82) and HB0025 (83) incorporating the extracellular domain 2 of VEGFR1 (a “VEGF trap”) at the N-termini of heavy chains in an anti-PD-L1 mAb scaffold (**Figure 1B**). Recently, promising preliminary results of Phase 2 trials have been released for both candidates in front-line treatment of NSCLC (NCT06746870) and endometrial cancer patients (NCT06758557) (84), respectively.

At least ten other VEGF x PD-(L)1 bi- and trispecific antibodies are at earlier clinical stage (**Table 1**). Combinations of three different binding moieties in a single molecule may offer a variety of new therapeutic options (85), such as simultaneously blocking two immune checkpoint inhibitors on T cells or removing two soluble factors from the TME. Examples of such trispecifics in clinical trials include three VEGF x PD-1 x CTLA-4 coined as CS2009 (86), HC010 (87) and GB268 (88), as well as the VEGF x PD-L1 x TGF-β DR30206 (89).

## Safety of VEGF x PD-(L)1 bispecific antibodies

Apparently, treatment-related adverse event (TRAEs) are not more frequent using bispecifics that combinations of anti-VEGF and anti- PD-(L)1 antibodies. In the first ivonescimab phase II trial (90), it was even reported a lower rate of grade 3–4 TRAEs in patients treated with ivonescimab plus chemotherapy (26.5% versus 58.5%) compared with the ABCP group (atezolizumab plus bevacizumab plus chemotherapy) of a combination study (65). The immune-related adverse event (irAEs) were generally low-grade and ≥ 3 grade irAEs occurred in 2.4% of patients. In the phase 3 study Harmoni-2 (70), grades 3–4 TRAEs occurred in 29% patients with ivonescimab and 16% patients with pembrolizumab, and ≥ 3 grade irAEs were observed in 7% and 8% of patients, respectively. In the case of BNT327 (91), grade ≥ 3 TRAEs occurred in a higher percentage of patients (54.7%), while incidence of grade ≥ 3 irAEs were in line with the previous studies on ivonescimab (4.7% patients). Incidence of severe adverse effects in patients receiving SSGJ-707 was similar to the observed in Harmoni-2 (24.1% experienced grade ≥ 3 TRAEs) (76).

Another issue is the potential application of antiangiogenic agents in squamous NSCLC (sq-NSCLC) patients, whose lesions may exhibit extensive necrosis resulting in hemorrhage. Indeed, the incidence of major hemorrhagic events in a phase II trial with bevacizumab precluded its subsequent development in these patients. Despite this theoretical concern, rates of serious bleeding events in the ivonescimab phase II trial (90) and HARMONi-2 (70) were low overall and safety was comparable between patients with sq- and nsq-NSCLC (after exclusion of those with severe bleeding tendency). These results could be attributed to the shorter half-life of ivonescimab (6–7 days) compared with bevacizumab (20 days), which may permit restoration of VEGF levels between administrations (92). Similarly, no special incidence of TRAEs in sq-NSCLC patients treated with SSGJ-707 was reported (76). Not surprisingly, all global phase 3 trials ongoing in NSCLC patients include squamous and non-squamous cohorts.

## Biomarkers for VEGF x PD-(L)1 bispecific antibodies: a pending issue

The biomarkers required to identify patients that could benefit from treatment with VEGF x PD-(L)1 bi- or trispecific antibodies, beyond PD-L1 expression, remain to be investigated. Up to date, only PD-L1 has been explored as a predictive biomarker of response in clinical trials. In the first ivonescimab phase II trial (90), ORR was higher in the participants with PD-L1 tumor proportion score (TPS) of 1%–49% and ≥50% compared with <1%, but results were not conclusive given that the small number of patients in each subgroup. In patients treated with SSGJ-707, ORR were 57% and 69% in patients with PD-L1 TPS 1%-49% and ≥ 50%, respectively (76). In the trial with BNT327/PM8002 in NSCLC, the PD-L1 TPS<1% group had an ORR of 35.7%, increasing to 56.5% in the TPS 1-49% group and 92.3% in the TPS ≥50% group (91). These results should be confirmed by larger studies and be analyzed in the context of new biomarkers addressing the angiogenic status of each tumor, including for example angiogenic signatures, hypoxia scores, or scRNA-seq–derived endothelial subsets. Non-invasive methods to quantify tumor hypoxia, such as PET imaging, could be used to longitudinally monitor patients receiving VEGF x PD-(L)1 treatments.

## Conclusions

The promising early results with VEGF x PD-(L)1 bispecific antibodies has led to renewed interest in targeting angiogenesis as a therapeutic approach. These antibodies have the potential to become a standard of care not only for NSCLC, but also for multiple solid tumors, if ongoing clinical trials are up to expectations. To fulfil their potential it will be necessary to select patients who may benefit, identified by appropriate biomarkers of response and resistance. Mature OS data and validation across different populations in real-world studies will be critical for these agents to displace conventional monospecific antibodies and establish VEGF x PD-(L)1 bispecifics as a new cornerstone of cancer immunotherapy.

## Data Availability

The original contributions presented in the study are included in the article/supplementary material. Further inquiries can be directed to the corresponding authors.
